# Quality of life and sexual health in patients with hidradenitis suppurativa^☆^^☆☆^

**DOI:** 10.1016/j.ijwd.2017.10.007

**Published:** 2018-02-01

**Authors:** A. Alavi, D. Farzanfar, T. Rogalska, M.A. Lowes, S. Chavoshi

**Affiliations:** a Department of Medicine (Dermatology), University of Toronto, Toronto, Ontario, Canada; b University Health Network, University of Toronto, Toronto, Ontario, Canada; c Department of Family Medicine, University of Ottawa, Ottawa, Ontario, Canada; d The Rockefeller University, New York, United States; e Department of Psychology, York University, Toronto, Ontario, Canada; f Division of Dermatology, Women's College Hospital, Toronto, Canada

## Abstract

**Background:**

Hidradenitis suppurativa (HS) is a chronic, recurrent, debilitating follicular disease. The effect of HS on physical and psychological aspects of sexual function is not well understood.

**Objective:**

The objective of this study is to investigate the contribution of sexual dysfunction to the quality of life (QoL) of patients with HS and to investigate the extent to which sexual health predicts the QoL in these patients.

**Methods:**

This is an observational cross-sectional study of 50 patients with HS and 50 healthy volunteers who completed questionnaires to measure QoL and sexual functioning using four validated tools.

**Results:**

Male patients experienced higher sexual dysfunction and a reduced quality of sexual life, while female patients reported higher sexual distress, compared with control groups. In male patients, sexual QoL and erectile dysfunction predicted a 72% decline in QoL. In female patients, sexual distress and sexual dysfunction predicted 46% variability in QoL index scores, beyond the effects of disease severity.

**Conclusion:**

Disruptions to sexual functioning greatly contribute to QoL impairments in patients with HS regardless of genital lesions. Health care professionals should inquire about and pay close attention to sexual health concerns in patients with HS.

## Introduction

Hidradenitis suppurativa (HS) significantly impairs patients’ quality of life (QoL; Jemec and Wulf, 1996, Jemec et al., 1996, von der Werth and Jemec, 2001) with greater disability than many other dermatological conditions (Balieva et al., 2017, Wolkenstein et al., 2007). HS is characterized by recurrent, painful, inflammatory nodules in intertriginous regions of the body such as the axillary, inguinal, inframammary and anogenital regions (Deckers and Kimball 2016). These symptoms, exacerbated by malodourous discharge and pain (Esmann and Jemec 2011), hinder patients’ sexual function and intimacy in interpersonal relationships, particularly in the younger population (Sampogna et al. 2017). Many patients with HS experience feelings of embarrassment, shame, diminished self-worth, and isolation as a result of the associated smell, itching, pain, and scarring (Matusiak et al. 2010). A two-fold increase in risk of completed suicides in patients with HS has been recently reported, although the underlying contributing factors are yet to be explored (Garg et al. 2017).

Sexual health encompasses both the physical component of sexual functioning as well as the psychological component of an individual’s subjective experience of sexuality (Verschuren et al. 2010). Overall genital involvement in any dermatological disease is associated with higher sexual distress (Sampogna et al., 2017, Van De Nieuwenhof et al., 2010). Therefore, it is not surprising that HS disproportionately affects patients’ sexual health (Janse et al. 2017), which is an important determinant of overall QoL (Verschuren et al. 2010). Sexual health impairment is strongly linked with psychological disturbances that lead to depression, anxiety, and suicidal ideation in patients with HS, even after controlling for other comorbidities such as obesity, diabetes, and thyroid disorders (Deckers and Kimball, 2016, Sampogna et al., 2017).

Although the impact of HS on patients’ sexual health has been gaining more attention in recent years, the literature on this topic remains limited. Our objective was to evaluate the extent to which impairments in sexual health adversely affect QoL in patients with HS compared with healthy individuals. Given that HS disproportionately affects female patients, sex differences in the level of QoL impairment were also explored in this study. We hypothesized that both the physical and psychological aspects of sexual functioning predict QoL in patients with HS. We confirmed that HS significantly reduced the QoL of both male and female patients and found that sexual dysfunction was a major contributor that could predict this negative impact on QoL.

## Methods

### Study design and participants

This observational cross-sectional study included 50 patients with HS and 50 controls who were matched for age and sex. The study was approved by the Women’s College Hospital research ethics board in Toronto, Ontario, Canada. The inclusion criteria for the HS group included the ability to give informed consent, > 18 years of age, and a diagnosis of HS confirmed by a dermatologist. Disease severity was assessed with the Hurley staging system by a dermatologist during the clinic visit. Eligible patients with HS who presented at the Women’s College Hospital or the Richmond Hill Dermatology Clinic between October 2015 to 2016 were recruited to complete self-administered paper questionnaires.

The control group was composed of healthy individuals or individuals who accompanied the patient and who were matched for age and sex on the basis of self-reports. The individuals in the control group did not have HS or any known medical conditions, including HS comorbidities. Patients or healthy volunteers who had any other chronic dermatologic conditions during the past year or any malignant, psychiatric, or hormonal disorders were excluded.

### Quality of life and sexual health questionnaires

The primary outcomes of interest were overall QoL and sexual functioning. Four validated sexual health questionnaires were used to qualitatively assess the physical and psychological aspects of sexual functioning. The validated instruments that were utilized to evaluate QoL and sexual functioning included the Dermatology Life Quality Index (DLQI), Sexual Quality of Life Questionnaire for Use in Men (SQoLM), International Index of Erectile Dysfunction (IIEF), Female Sexual Function Index (FSFI), and Female Sexual Distress Scale – Revised (FSDS-R).

The DLQI is a validated, 10-item questionnaire that evaluates the QoL of patients with a dermatological disease. The questionnaire encompasses six domains: symptoms and feelings, daily activities, leisure, work and school, personal relationships, and disease management. There is a question on sexual health in the DLQI, but the DLQI has been validated for use as a single 10-question tool. The total score is calculated by summing the scores of each question (minimum = 0, maximum = 30). The higher the DLQI score, the more QoL is impaired (Finlay and Khan 1994).

The SQoLM is a self-administered instrument that is used to assess the sexual QoL in men and was completed by male participants. The instrument contains 11 items, each on a six-point response scale. The total scores are calculated in accordance with the SQoLM scoring manual (minimum = 0, maximum = 100). Higher scores denote a higher QoL (Abraham et al. 2008).

The IIEF was completed also by the male participants. The IIEF is a 15-item questionnaire that assesses five domains of male sexual function: erectile function, orgasmic function, sexual desire, intercourse satisfaction, and overall satisfaction. The scores for individual items are summed in accordance with the scoring manual (minimum = 5, maximum = 75). Higher scores indicate better sexual function (Rosen et al. 2000).

The FSDS-R is a questionnaire that was completed by the female participants. The FSDS-R questionnaire is composed of 13 items that measure sexually related distress in women. The FSDS-R differs from the FSDS in that it includes an item on distress that is related to low sexual desire. The minimum possible score is 0, and the maximum possible score is 52. An FSDS-R score of ≥ 11 indicates female sexual dysfunction. Higher scores indicate greater sexual dysfunction (Derogatis et al. 2008).

The Female Sexual Function Index (FSFI) was completed also by female subjects and consists of a 19-item questionnaire that assesses six domains of female sexual function: sexual desire, arousal, lubrication, orgasm, satisfaction, and pain. Scores are calculated by adding the six domain scores (if one or more items of a domain are missing, a domain score is not calculated). The minimum possible score is 2, and the maximum possible score is 36. FSFI total scores of ≤ 26.55 indicate sexual dysfunction. Higher scores indicate better sexual functioning (Rosen et al., 2000, Wiegel et al., 2005).

### Statistical analysis

Outcome variable scores were evaluated in accordance with the individual instrument criteria (ie, DLQI, SWoL-M, IIEF, FSFI, and FSDS-R). The statistical analysis was performed using SPSS Statistics, Version 23 (IBM, Armonk, NY). Continuous variables were described as mean ± standard deviation, and discontinuous variables were described by total frequencies and percentages of each modality. The differences between patients in the HS and control groups were evaluated with a χ^2^ test for categorical variables. Continuous variables were compared with independent Student’s *t* tests. Pearson correlation tests were conducted to assess the effect size for the association between variables. Two-sided statistical significance was assessed at *p* < .05.

A hierarchical regression analysis was used to examine the influence of predictor variables sequentially (Cohen 2008), such that the relative importance of sexual health in predicting QoL could be deciphered, over and above the contribution of disease severity. This analysis was performed separately for male and female patients due to the differences in sexual function instruments.

Hurley stage and the current number of lesions were used to account for disease severity. In simple terms, hierarchic regression is the same as multiple regression when performed in separate steps, such that certain predictors have the first chance to explain the outcome of interest. Any additional predictors have to provide a useful explanation or predictive ability beyond what has already been accounted for by the preceding steps. We used this method to show that the sexual distress and dysfunction scales predict impairment in the QoL of patients beyond what can already be explained using the extent of their disease progression and current number of lesions.

## Results

**Fig. 1:**
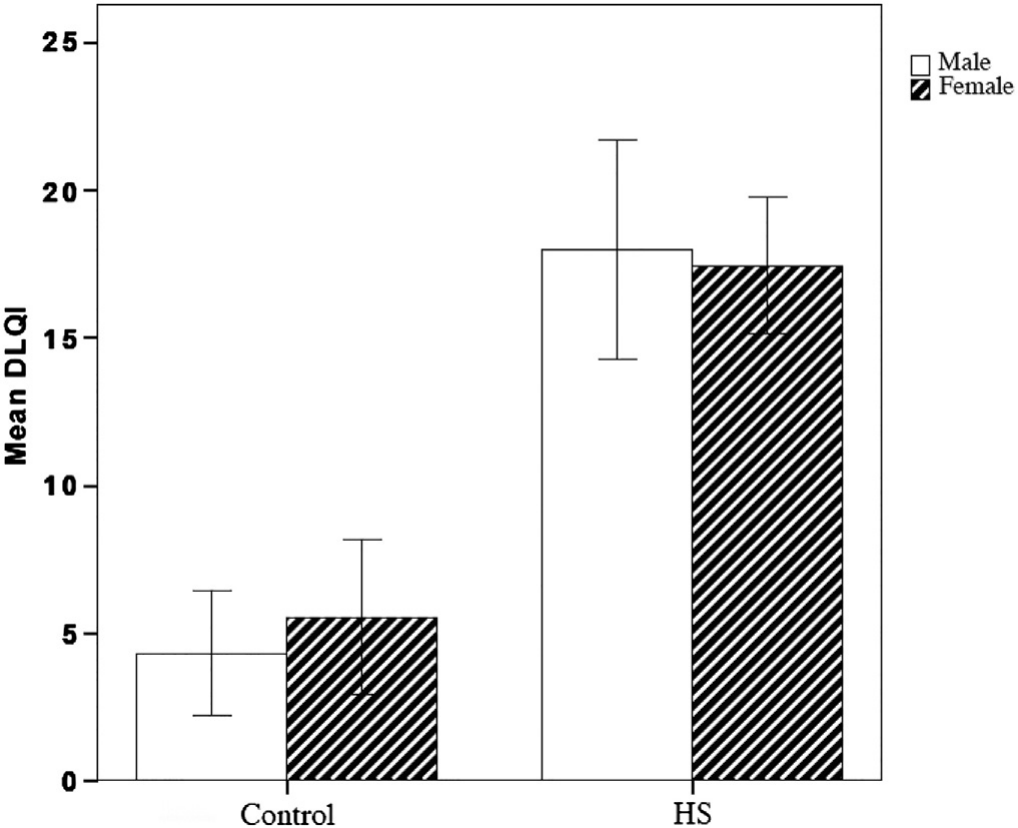
Quality of Life Impairment index across the sexes between groups of patients with hidradenitis suppurativa (HS) and control subjects. HS > Control, *p* < .0001. Lower DLQI value denotes higher QoL.

Patients with HS had a significantly lower QoL as measured with the DLQI compared with the control group (*p* < .0001; Fig. 1). The DLQI scores of male and female patients with HS did not differ, but they were significantly different when compared with those of the same sex control group.

The demographic characeteristics of both patients with HS and healthy controls were very similar in the study (Table 1). In the HS cohort, the female-to-male ratio was 3:1.5 and the mean age was 34 years for female and 40 years for male patients. The additional group mean difference analyses on basic demographic information such as age, sex, relationship status, and body mass index are provided in Table 2.

**Table 1:** Subgroup analysis

Variables/Subgroups	Patients with HS (n = 50)	Control patients (n = 50)	*p*-value
**Sex**			.41
Female	33	28	
Male	17	22	
**Age**	35.98 ± 13.62	39.80 ± 11.80	.14
**Body mass index**	30.70 ± 7.71	25.45 ± 5.57	< .001
**Relationship status**			.45
Married/common law	18	29	
Single	24	15	
Separated	4	3	

HS, hidradenitis suppurativa

**Table 2:** Demographic and clinical characteristics for patients with HS

Variables	All patients with HS (n = 50)	Female patients with HS (n = 33)	Male Patients with HS (n = 17)
**Age**	35.98 ± 13.62	33.67 ± 12.16	40.47 ± 15.49
**Age of HS onset**	22.33 ± 12.41	19.19 ± 8.91	29.75 ± 5.24
**Years to diagnosis**	9.50 ± 8.74	8.40 ± 8.88	12.42 ± 7.95
**Current number of lesions**	13.90 ± 17.83	11.97 ± 12.04	18.62 ± 27.51
**Body mass index**	30.70 ± 7.71	31.20 ± 8.79	29.75 ± 5.24
**Race**			
Caucasian	38	25	13
Black	6	6	0
Other	4	2	2
**Relationship status**			
Married	18	11	7
Single	24	17	7
Separated	4	3	1
**First lesion location**			
Groins	10	7	3
Axillae	9	6	3
Genitals/Pelvic	5	3	2
Trunk	10	3	7
**Alcohol consumption**			
Yes	30	21	9
No	15	12	3
**Smoking status**			
Current smoker	17	11	6
Past smoker	12	6	6
Nonsmoker	20	16	4
**Medical history**			
Diabetes mellitus	4	4	0
Hypertension	4	3	1
Dyslipidemia	6	3	3
PCOS	2	2	0
Acne	18	12	7
Pilonydal cyst	6	1	3
Thyroid disease	2	2	0
Psychiatric	16	12	4
Crohn’s disease	2	2	0
Cancer	2	1	1
None	29	14	10
**Surgical history**			
Yes	18	13	5
No	29	20	9
**Family history of HS**			
Yes	9	5	4
No	36	27	9
**First HS diagnosis**			
Dermatologist	24	16	8
Family Physician	10	9	1
Surgeon	7	5	2
Other	2	1	1
Patient	3	2	1
**Genital lesions**			
Yes	27	21	6
No	14	7	7
**Hurley Stage**			
Stage II	24	17	7
Stage III	23	15	8

HS, hidradenitis suppurativa; PCOS, polycystic ovary syndrome

**Fig. 2:**
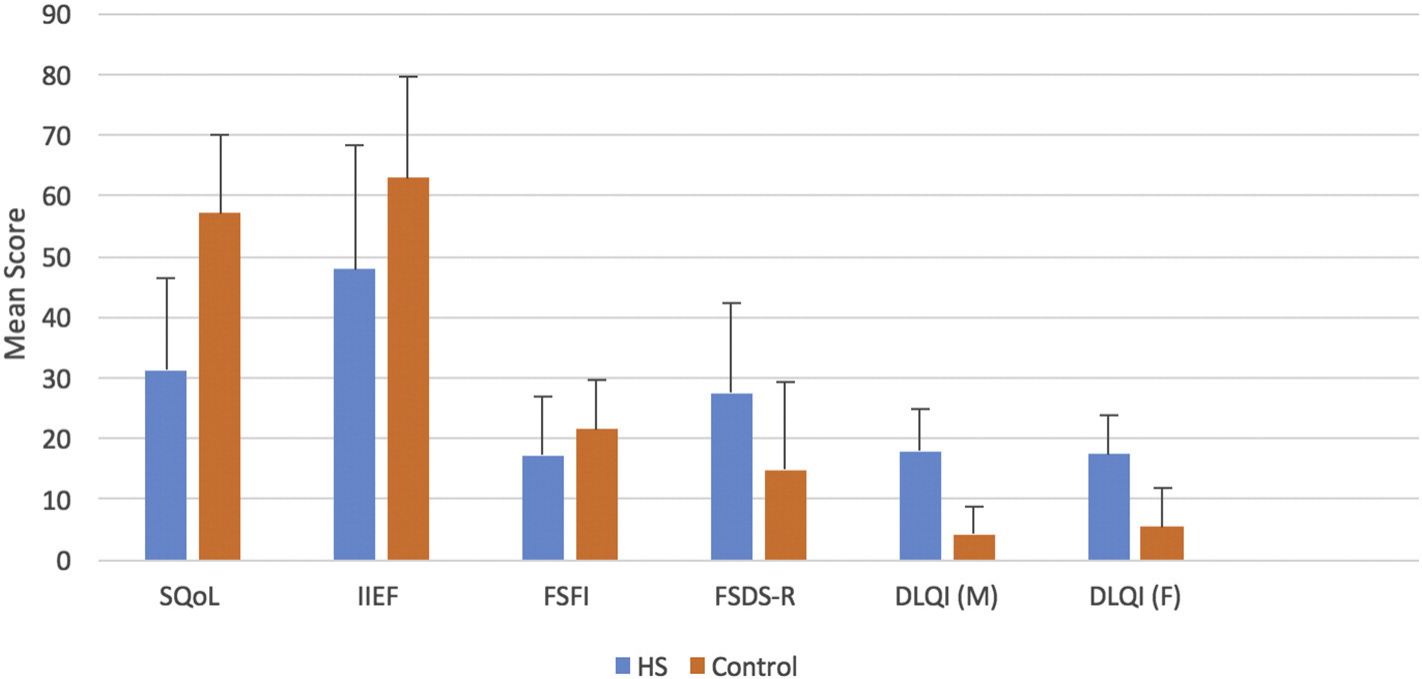
Summary of differences in mean sexual function measures (mean score) between patients with hidradenitis suppurativa and the control group. Sexual Quality of Life Questionnaire for Use in Men (SQoLM) (*p* < .0001), International Index of Erectile Dysfunction (IIEF) (*p* = .019), Female Sexual Function Index (FSFI) (not significant, *p* = .075), Female Sexual Distress Scale - Revised (FSDS-R) (*p* = .002), Dermatology Life Quality Index (DLQI) (male patients, M) (*p* < .001) and DLQI (female patients, F) (*p* < .001).

**Fig. 3:**
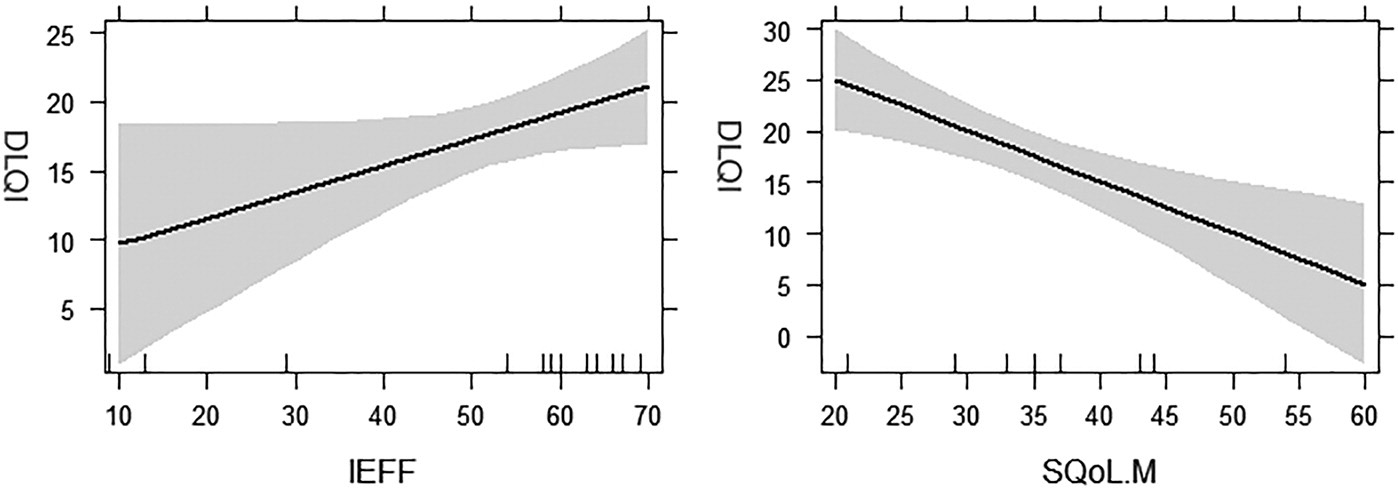
Regression effects plots for quality of life and measures of sexual functioning in male patients with hidradenitis suppurativa. These plots shows the relationship between quality of life (Dermatology Life Quality Index, DLQI) and International Index of Erectile Dysfunction (IEEF), and Sexual Quality of Life Questionnaire for Use in Men (SQoLM) in men when controlling for disease progression and current number of lesions. The IIEF was not a significant predictor of Dermatology Life Quality Index.

The mean differences for the four questionnaires assessing sexual function are shown in Figure 2. When compared with the male control group, male patients with HS had on average a lower sexual SQoLM (*p* < .0001) and IIEF scores (*p* = .019). Figure 3 depicts the relationship between DLQI and male sexual function measures.

To establish a baseline correlation, the association between Item 9 of the DLQI, which asks patients about the extent of sexual difficulties caused by the skin, and the four sexual health measures were examined. Item 9 was strongly and significantly correlated with FSDS-R (*r* = .614; *p* < .001) and SQoLM (*r* = –0.64; *p* = .001). There was no correlation between Item 9 and IIEF (*r* = –0.25; *p* = .25) and FSFI (*r* = –0.19; *p* = .21) (Table 3).

**Table 3:** Pearson Correlation matrix between sexual health measures, age, and severity

Variables	Age	Age of HS onset	Time to diagnosis	Number of lesions	Hurley Stage
**Female**					
FSFI	.13	–.19	.22	.03	.01
FSDS-R	.11	.33	.00	.03	.09
**Male**					
SQoLM	–.13	–.44	.39	–.23	–.28
IEFF	–.39^a^	–.61^a^	–.02	.14	–.07

FSDS-R, Female Sexual Distress Scale – Revised; FSFI, Female Sexual Function Index; HS, hidradenitis suppurativa; IEFF, International Index of Erectile Dysfunction; SQoLM, sexual quality of life in male patients

a *p* < .05

**Fig. 4:**
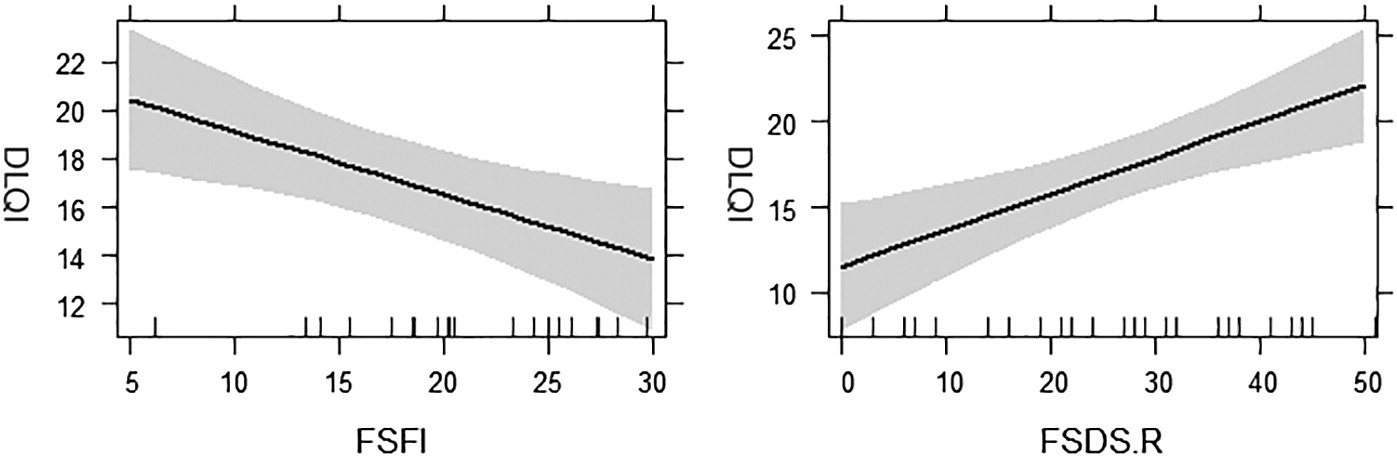
Regression effects plots for quality of life and measures of sexual functioning in female patients with hidradenitis suppurativa. These plots show the relationship between quality of life (Dermatology Life Quality Index, DLQI) and Female Sexual Function Index (FSFI) and Female Sexual Distress Scale-Revised (FSDS-R).

Female patients had significantly higher distress related to sexual function as measured with the FSDS-R compared with healthy female volunteers (*p* = .002). No significant difference was found between female patients with HS and the control group with regard to sexual functioning as measured by the FSFI (*p* = .075). Figure 4 depicts the relationship between DLQI and female sexual function measures. Interestingly, the presence of genital lesions in both male and female patients did not correlate with DLQI changes or any sexual function measures.

A hierarchical regression analysis showed that 72% of variation in DLQI scores in male patients can be explained by a linear model with IIEF, SQoL, and disease progression as predictors (Table 4). The Hurley staging and number of lesions explained 39% of variance in DLQI, and the IIEF and SQoL together explained an additional 42% of variance. Similarly, female sexual distress and dysfunction accounted for changes in QoL and explained more than 46% of the variance (Adjusted R^2^, .46) over and above what could be accounted for by Hurley stage or current number of lesions.

**Table 4:** Hierarchical regression analysis on Dermatology Life Quality Index in male and female patients

	Blocks	Variables	*B*	SE B	*β*	*R*^*2*^	Δ*R*^*2*^	ΔF
**Female**								
	Block 1					0.05	0.11	1.76
		Hurley Stage	4.24	2.37	0.33			
		Number of Lesions	0.01	0.097	0.028			
	Block 2					0.46	0.42	11.32*
		FSDS-R	0.21*	0.06	0.49			
		FSFI	–0.26*	0.09	–0.40			
**Male**								
	Block 1					0.27	0.39	3.24
		Hurley Stage	–3.28	3.40	–0.24			
		Number of Lesions	0.16	0.06	–0.64			
	Block 2					0.72	0.42	8.89*
		IEFF	0.19	0.08	0.58			
		SQoLM	–0.49*	0.13	–1.06			

IEFF, International Index of Erectile Dysfunction; FSDS-R, Female Sexual Distress Scale – Revised; SQoLM, sexual quality of life in male patients

* p < .01

Overall, these results show that both male and female patients with HS experienced a negative impact on QoL and that sexual dysfunction is an important contributor to this impaired QoL.

## Discussion

Sexual health is an integral part of general health and an important measure of QoL. The universal importance of healthy sexual function for physical and mental wellbeing is well studied and includes effects on psychological, social, and emotional measures of quality of life. (Dunn et al., 1999, Ermertcan, 2009) As highlighted by the World Health Organization’s statement that “there is no health without mental health” (Prince and Prince 2001), the contribution of mental health to the physical burden of disease must not be undervalued. Sexual health is an important component of mental health, and sexual dysfunction is a multifaceted problem that is commonly underdiagnosed and undervalued.

The effect of HS on QoL and the correlation with disease severity has been shown in previous studies (Alavi et al. 2015). Impairment of sexual life was previously assessed in a multicenter study of 3485 European patients, with Item 9 of the DLQI score used as an indicator of the sexual impact of dermatological conditions (Sampogna et al. 2017). The study showed that 23% of patients reported sexual problems, with higher impairment noted in patients with HS (Sampogna et al. 2017). A later study by Kurek et al. (2012) studied 85 volunteer participants, and the results showed that all patients with HS suffered from sexual dysfunction and sexual distress compared with the matched control subjects. Moreover, sexual distress was particularly high in female patients with HS compared with male patients.

In the current study, we used four clinical instruments that are commonly used for the assessment of sexual health as well as the DLQI as a general measure of dermatological QoL. Our findings provide strong data for sexual health impairment and reduced QoL in patients with HS. Male patients experience significant sexual dysfunction and reduced sexual QoL, and women report significant sexual distress. Our study indicates that sexual health alone can largely account for the impaired QoL in patients with HS. This aspect of QoL may be captured with generic tools, particularly in severe cases of sexual dysfunction that are observed frequently among patients with HS.

It is plausible that HS affects sexual life as a result of its involvement of intimate areas of the body, the presence of pain, and associated malodor. Janse et al. (2017) showed that sexual health impairment in patients with HS was linked to anogenital involvement, early onset of HS, and disease severity. Surprisingly, our study did not show that the presence of genital lesions in both male and female patients correlated with DLQI changes or any sexual function measures.

Addressing the sexual health concerns of patients depends on the clinician’s ability to communicate this sensitive issue, which may not be adequately broached in a fast-paced dermatology practice. It may be challenging for both patients and physicians to discuss sexual health directly. The knowledge of these disruptions to sexual functioning in patients with HS should encourage physicians to pay close attention to patients’ sex-related concerns. It is also important to connect patients with HS with appropriate psychological and counseling networks to minimize the impact of poor self-image, low self-esteem, depression, and anxiety (Balieva et al. 2017) on the quality of sexual life in these patients. Future studies should focus on identifying patients who are at a higher risk for sexual health impairments and explore the link between sexual functioning and overall QoL in relation to specific HS phenotypes and clinical severity.

## Limitations and future directions

Given that there are few male patients from whom we obtained data, our analysis likely does not have enough power to detect small and medium effect sizes. Although the hierarchical regression analysis was repeated with data from male patients, the results should be interpreted cautiously and only through the lens of an exploratory study. Our sample size may have also hindered the ability to detect differences in FSFI scores among women with HS and the control group.

Another limitation is the inability to compare sexual functioning between the two sexes due to the use of sex-specific sexual functioning instruments. Therefore, future studies should focus on utilizing a common set of measures for sexual functioning in male and female patients to compare the differences in sexual impairment due to HS directly.

A further limitation of the results of this study and similar studies of QoL outcomes in patients with HS is the lack of an HS-specific QoL instrument that can capture the symptoms most responsible for sexual dysfunction. The DLQI score reflects impairement in overall QoL; however, our study may suggest that sexual dysfunction can be reflected to an adequate degree. We believe that this is due to an indirect association between sexual function and overall QoL, which further strengthens the observed relationship in diseases that lead to severe impairements in any one aspect of OoL, in this case sexual health.

## Conclusions

The link between HS, sexual health, and patient wellbeing has often been underappreciated. The results of this study demonstrate a significant impact on sexual function in both female and male patients with HS in a North American population, regardless of whether the lesions were in genital areas. Given the significant role of sexual wellbeing in both mental and physical health, dermatologists should be aware of the scope of impairment that this disease may have for their patients even without the presence of genital lesions. A simple questionnaire can be used to screen for sexual dysfunction in an office setting, and physicians should pay close attention to counselling, communication, and appropriate referral for these patients.

The results of this study reinforce the need to take sexual health into account when assessing disease severity, response to treatment, and patient goals of care.

## Appendix A Supplementary Data

Supplementary material. Image 1
