# Supplementary material for: Quality of life and sexual health in patients with hidradenitis suppurativa
Source: Int J Womens Dermatol. 2018 Feb 1;4(2):74–9. doi: 10.1016/j.ijwd.2017.10.007 (PMC6047191; doi:10.1016/j.ijwd.2017.10.007)
Supplement: Supplementary file 1 — Supplementary material. [file mmc1.docx]

**QUESTIONNAIRE – STUDY GROUP**

**STUDY TITLE:** Quality of life and sexual health in patients with hidradenitis suppurativa

**SUBJECT CODE:**

Your responses will be kept completely confidential.

DEMOGRAPHIC INFORMATION:

1. Age (years):
2. Sex (M/F):
3. Height (cm):
4. Weight (kg):
5. Relationship status:

COURSE OF DISEASE:

1. Age at hidradenitis suppurativa diagnosis:
2. Where on your body did HS first appear? :
3. Current genital involvement (Y/N):
4. Previous genital involvement (Y/N):
5. Do you currently smoke, or have you smoked in the last year? (Y/N):

DERMATOLOGY LIFE QUALITY INDEX (DLQI):

The aim of this section of the questionnaire is to measure how much your skin condition has affected your life **OVER THE LAST YEAR**. Please check one box for each question. You may choose not to answer any question for any reason, and still return the questionnaire.

1. Over the last year, how **itchy, sore, painful, or stinging** has your skin been?

- Very much
- A lot
- A little
- Not at all

1. Over the last year, how **embarrassed or self-conscious** have you been because of your skin?

- Very much
- A lot
- A little
- Not at all

1. Over the last year, how much has your skin interfered with you going **shopping** or looking after your **home** or **garden**?

- Very much
- A lot
- A little
- Not at all
- Not relevant

1. Over the last year, how much has your skin influenced the **clothes** that you wear?

- Very much
- A lot
- A little
- Not at all
- Not relevant

1. Over the last year, how much has your skin affected any **social** or **leisure** activities?

- Very much
- A lot
- A little
- Not at all
- Not relevant

1. Over the last year, how much has your skin made it difficult for you to do any **sport**?

- Very much
- A lot
- A little
- Not at all
- Not relevant

1. Over the last year, has your skin prevented you from **working** or **studying**?

- Yes
- No
- Not relevant

If ‘No’, over the last year how much has your skin been a problem at **work** or **studying**?

- A lot
- A little
- Not at all

1. Over the last year, how much has your skin created problems with your **partner** or any of your **close friends** or **relatives**?

- Very much
- A lot
- A little
- Not at all
- Not relevant

1. Over the last year, how much has your skin caused **sexual difficulties**?

- Very much
- A lot
- A little
- Not at all
- Not relevant

1. Over the last year, how much of a problem has the **treatment** for your skin been, for example by making your home messy, or by taking up time?

- Very much
- A lot
- A little
- Not at all
- Not relevant

For the following section, please complete questionnaires for **males** or **females**, respectively.

In answering these questions, the following definitions apply:

Sexual activity can include caressing, foreplay, masturbation, and intercourse.

Sexual stimulation includes situations like foreplay with a partner, self-stimulation (masturbation), or sexual fantasy.

SEXUAL QUALITY OF LIFE (**MALE**)

This questionnaire consists of a set of questions, each asking about thoughts and feelings that you may have about your sex life. Please answer based on your thoughts and feelings **OVER THE LAST YEAR**. You may choose not to answer any question for any reason, and still return the questionnaire.

Please rate each one according to how much you agree or disagree with the statement, by circling **one** of six response choices.

1. When I think about my sexual life, I feel frustrated

| Completely Agree | Moderately Agree | Slightly Agree | Slightly Disagree | Moderately Disagree | Completely Disagree |
| --- | --- | --- | --- | --- | --- |
| 1 | 2 | 3 | 4 | 5 | 6 |

1. When I think about my sexual life, I feel depressed

| Completely Agree | Moderately Agree | Slightly Agree | Slightly Disagree | Moderately Disagree | Completely Disagree |
| --- | --- | --- | --- | --- | --- |
| 1 | 2 | 3 | 4 | 5 | 6 |

1. When I think about my sexual life, I feel like less of a man

| Completely Agree | Moderately Agree | Slightly Agree | Slightly Disagree | Moderately Disagree | Completely Disagree |
| --- | --- | --- | --- | --- | --- |
| 1 | 2 | 3 | 4 | 5 | 6 |

1. I have lost confidence in myself as a sexual partner

| Completely Agree | Moderately Agree | Slightly Agree | Slightly Disagree | Moderately Disagree | Completely Disagree |
| --- | --- | --- | --- | --- | --- |
| 1 | 2 | 3 | 4 | 5 | 6 |

1. When I think about my sexual life, I feel anxious

| Completely Agree | Moderately Agree | Slightly Agree | Slightly Disagree | Moderately Disagree | Completely Disagree |
| --- | --- | --- | --- | --- | --- |
| 1 | 2 | 3 | 4 | 5 | 6 |

1. When I think about my sexual life, I feel angry

| Completely Agree | Moderately Agree | Slightly Agree | Slightly Disagree | Moderately Disagree | Completely Disagree |
| --- | --- | --- | --- | --- | --- |
| 1 | 2 | 3 | 4 | 5 | 6 |

1. I worry about the future of my sexual life

| Completely Agree | Moderately Agree | Slightly Agree | Slightly Disagree | Moderately Disagree | Completely Disagree |
| --- | --- | --- | --- | --- | --- |
| 1 | 2 | 3 | 4 | 5 | 6 |

1. When I think about my sexual life, I am embarrassed

| Completely Agree | Moderately Agree | Slightly Agree | Slightly Disagree | Moderately Disagree | Completely Disagree |
| --- | --- | --- | --- | --- | --- |
| 1 | 2 | 3 | 4 | 5 | 6 |

1. When I think about my sexual life, I feel guilty

| Completely Agree | Moderately Agree | Slightly Agree | Slightly Disagree | Moderately Disagree | Completely Disagree |
| --- | --- | --- | --- | --- | --- |
| 1 | 2 | 3 | 4 | 5 | 6 |

1. When I think about my sexual life, I worry that my partner feels hurt or rejected

| Completely Agree | Moderately Agree | Slightly Agree | Slightly Disagree | Moderately Disagree | Completely Disagree |
| --- | --- | --- | --- | --- | --- |
| 1 | 2 | 3 | 4 | 5 | 6 |

1. When I think about my sexual life, I feel like I have lost something

| Completely Agree | Moderately Agree | Slightly Agree | Slightly Disagree | Moderately Disagree | Completely Disagree |
| --- | --- | --- | --- | --- | --- |
| 1 | 2 | 3 | 4 | 5 | 6 |

INTERNATIONAL INDEX OF ERECTILE FUNCTION (IIEF) (**MALE**)

The following questions ask about the effects your erection problems have had on your sex life **OVER THE LAST YEAR**. Please mark only one answer per question. You may choose not to answer any question for any reason, and still return the questionnaire.

1. Over the last year, how often were you able to get an erection during sexual activity?

| No sexual activity | Almost never or never | A few times (much less than half the time) | Sometimes (about half the time) | Most times (much more than half the time) | Almost always or always |
| --- | --- | --- | --- | --- | --- |
| 0 | 1 | 2 | 3 | 4 | 5 |

1. Over the last year, when you had erections with sexual stimulation, how often were your erections hard enough for penetration?

| No sexual activity | Almost never or never | A few times (much less than half the time) | Sometimes (about half the time) | Most times (much more than half the time) | Almost always or always |
| --- | --- | --- | --- | --- | --- |
| 0 | 1 | 2 | 3 | 4 | 5 |

1. Over the last year, when you attempted sexual intercourse, how often were you able to penetrate (enter) your partner?

| Did not attempt intercourse | Almost never or never | A few times (much less than half the time) | Sometimes (about half the time) | Most times (much more than half the time) | Almost always or always |
| --- | --- | --- | --- | --- | --- |
| 0 | 1 | 2 | 3 | 4 | 5 |

1. Over the last year, during sexual intercourse, **how often** were you able to maintain your erection after you had penetrated (entered) your partner?

| Did not attempt intercourse | Almost never or never | A few times (much less than half the time) | Sometimes (about half the time) | Most times (much more than half the time) | Almost always or always |
| --- | --- | --- | --- | --- | --- |
| 0 | 1 | 2 | 3 | 4 | 5 |

1. Over the last year, during sexual intercourse, **how difficult** was it to maintain your erection to completion of intercourse?

| Did not attempt intercourse | Extremely difficult | Very difficult | Difficult | Slightly difficult | Not difficult |
| --- | --- | --- | --- | --- | --- |
| 0 | 1 | 2 | 3 | 4 | 5 |

1. Over the last year, how many times have you attempted sexual intercourse?

| No attempts | One to two attempts | Three to four attempts | Five to six attempts | Seven to ten attempts | Eleven or more attempts |
| --- | --- | --- | --- | --- | --- |
| 0 | 1 | 2 | 3 | 4 | 5 |

1. Over the last year, when you attempted sexual intercourse, how often was it satisfactory for you?

| Did not attempt intercourse | Almost never or never | A few times (much less than half the time) | Sometimes (about half the time) | Most times (much more than half the time) | Almost always or always |
| --- | --- | --- | --- | --- | --- |
| 0 | 1 | 2 | 3 | 4 | 5 |

1. Over the last year, how much have you enjoyed sexual intercourse?

| No intercourse | No enjoyment | Not very enjoyable | Fairly enjoyable | Highly enjoyable | Very highly enjoyable |
| --- | --- | --- | --- | --- | --- |
| 0 | 1 | 2 | 3 | 4 | 5 |

1. Over the last year, when you had sexual stimulation **or** intercourse, how often did you ejaculate?

| No sexual stimulation or intercourse | Almost never or never | A few times (much less than half the time) | Sometimes (about half the time) | Most times (much more than half the time) | Almost always or always |
| --- | --- | --- | --- | --- | --- |
| 0 | 1 | 2 | 3 | 4 | 5 |

1. Over the last year, when you had sexual stimulation **or** intercourse, how often did you have the feeling of orgasm or climax?

| No sexual stimulation or intercourse | Almost never or never | A few times (much less than half the time) | Sometimes (about half the time) | Most times (much more than half the time) | Almost always or always |
| --- | --- | --- | --- | --- | --- |
| 0 | 1 | 2 | 3 | 4 | 5 |

1. Over the last year, how often have you felt sexual desire?

| Almost never or never | A few times (much less than half the time) | Sometimes (about half the time) | Most times (much more than half the time) | Almost always or always |
| --- | --- | --- | --- | --- |
| 1 | 2 | 3 | 4 | 5 |

1. Over the last year, how would you rate your level of sexual desire?

| Very low/none at all | Low | Moderate | High | Very high |
| --- | --- | --- | --- | --- |
| 1 | 2 | 3 | 4 | 5 |

1. Over the last year, how satisfied have you been with your overall **sex life**?

| Very dissatisfied | Moderately dissatisfied | About equally satisfied and dissatisfied | Moderately satisfied | Very satisfied |
| --- | --- | --- | --- | --- |
| 1 | 2 | 3 | 4 | 5 |

1. Over the last year, how satisfied have you been with you **sexual relationship** with your partner?

| Very dissatisfied | Moderately dissatisfied | About equally satisfied and dissatisfied | Moderately satisfied | Very satisfied |
| --- | --- | --- | --- | --- |
| 1 | 2 | 3 | 4 | 5 |

1. Over the last year, how would you rate your **confidence** that you could get and keep an erection?

| Very low | Low | Moderate | High | Very high |
| --- | --- | --- | --- | --- |
| 1 | 2 | 3 | 4 | 5 |

FEMALE SEXUAL FUNCTION INDEX (FSFI) (**FEMALE**)

This questionnaire consists of a set of questions, each asking about thoughts and feelings that you may have about your sex life. The statement may be positive or negative. Please answer based on your thoughts and feelings **OVER THE LAST YEAR**. You may choose not to answer any question for any reason, and still return the questionnaire.

Please rate each one according to how much you agree or disagree with the statement, by circling **one** of six response choices.

1. Over the last year, how **often** did you feel sexual desire or interest?

| Almost never or never | A few times (less than half the time) | Sometimes (about half the time) | Most times (more than half the time) | Almost always or always |
| --- | --- | --- | --- | --- |
| 1 | 2 | 3 | 4 | 5 |

1. Over the last year, how would you rate your **level** (degree) of sexual desire or interest?

| Very low or none at all | Low | Moderate | High | Very high |
| --- | --- | --- | --- | --- |
| 1 | 2 | 3 | 4 | 5 |

1. Over the last year, how **often** did you feel sexually aroused (“turned on”) during sexual activity or intercourse?

| No sexual activity | Almost never or never | A few times (less than half the time) | Sometimes (about half the time) | Most times (more than half the time) | Almost always or always |
| --- | --- | --- | --- | --- | --- |
| 0 | 1 | 2 | 3 | 4 | 5 |

1. Over the last year, how would you rate your **level** of sexual arousal (“turn on”) during sexual activity or intercourse?

| No sexual activity | Very low or none at all | Low | Moderate | High | Very high |
| --- | --- | --- | --- | --- | --- |
| 0 | 1 | 2 | 3 | 4 | 5 |

1. Over the last year, how **confident** were you about becoming sexually aroused during sexual activity or intercourse?

| No sexual activity | Very low or no confidence | Low confidence | Moderate confidence | High confidence | Very high confidence |
| --- | --- | --- | --- | --- | --- |
| 0 | 1 | 2 | 3 | 4 | 5 |

1. Over the last year, how **often** have you been satisfied with your arousal (excitement) during sexual activity or intercourse?

| No sexual activity | Almost never or never | A few times (less than half the time) | Sometimes (about half the time) | Most times (more than half the time) | Almost always or always |
| --- | --- | --- | --- | --- | --- |
| 0 | 1 | 2 | 3 | 4 | 5 |

1. Over the last year, how **often** did you become lubricated (“wet”) during sexual activity or intercourse?

| No sexual activity | Almost never or never | A few times (less than half the time) | Sometimes (about half the time) | Most times (more than half the time) | Almost always or always |
| --- | --- | --- | --- | --- | --- |
| 0 | 1 | 2 | 3 | 4 | 5 |

1. Over the last year, how **difficult** was it to become lubricated (“wet”) during sexual activity or intercourse?

| No sexual activity | Extremely difficult or impossible | Very difficult | Difficult | Slightly difficult | Not difficult |
| --- | --- | --- | --- | --- | --- |
| 0 | 1 | 2 | 3 | 4 | 5 |

1. Over the last year, how **often** did you maintain your lubrication (“wetness”) until completion of sexual activity or intercourse?

| No sexual activity | Almost never or never | A few times (less than half the time) | Sometimes (about half the time) | Most times (more than half the time) | Almost always or always |
| --- | --- | --- | --- | --- | --- |
| 0 | 1 | 2 | 3 | 4 | 5 |

1. Over the last year, how **difficult** was it to maintain your lubrication (“wetness”) until completion of sexual activity or intercourse?

| No sexual activity | Extremely difficult or impossible | Very difficult | Difficult | Slightly difficult | Not difficult |
| --- | --- | --- | --- | --- | --- |
| 0 | 1 | 2 | 3 | 4 | 5 |

1. Over the last year, when you had sexual stimulation or intercourse, how **often** did you reach orgasm (climax)?

| No sexual activity | Almost never or never | A few times (less than half the time) | Sometimes (about half the time) | Most times (more than half the time) | Almost always or always |
| --- | --- | --- | --- | --- | --- |
| 0 | 1 | 2 | 3 | 4 | 5 |

1. Over the last year, when you had sexual stimulation or intercourse, how **difficult** was it for you to reach orgasm (climax)?

| No sexual activity | Extremely difficult or impossible | Very difficult | Difficult | Slightly difficult | Not difficult |
| --- | --- | --- | --- | --- | --- |
| 0 | 1 | 2 | 3 | 4 | 5 |

1. Over the last year, how **satisfied** were you with your ability to reach orgasm (climax) during sexual activity or intercourse?

| No sexual activity | Very dissatisfied | Moderately dissatisfied | About equally satisfied and dissatisfied | Moderately satisfied | Very satisfied |
| --- | --- | --- | --- | --- | --- |
| 0 | 1 | 2 | 3 | 4 | 5 |

1. Over the last year, how **satisfied** have you been with the amount of emotional closeness during sexual activity between you and your partner?

| No sexual activity | Very dissatisfied | Moderately dissatisfied | About equally satisfied and dissatisfied | Moderately satisfied | Very satisfied |
| --- | --- | --- | --- | --- | --- |
| 0 | 1 | 2 | 3 | 4 | 5 |

1. Over the last year, how satisfied have you been with your sexual relationship with your partner?

| Very dissatisfied | Moderately dissatisfied | About equally satisfied and dissatisfied | Moderately satisfied | Very satisfied |
| --- | --- | --- | --- | --- |
| 1 | 2 | 3 | 4 | 5 |

1. Over the last year, how **satisfied** have you been with your overall sexual life?

| Very dissatisfied | Moderately dissatisfied | About equally satisfied and dissatisfied | Moderately satisfied | Very satisfied |
| --- | --- | --- | --- | --- |
| 1 | 2 | 3 | 4 | 5 |

1. Over the last year, how **often** did you experience discomfort or pain **during** vaginal penetration?

| Did not attempt intercourse | Almost always or always | Most times (more than half the time) | Sometimes (about half the time) | A few times (less than half the time) | Almost never or never |
| --- | --- | --- | --- | --- | --- |
| 0 | 1 | 2 | 3 | 4 | 5 |

1. Over the last year, how **often** did you experience discomfort or pain **following** vaginal penetration?

| Did not attempt intercourse | Almost always or always | Most times (more than half the time) | Sometimes (about half the time) | A few times (less than half the time) | Almost never or never |
| --- | --- | --- | --- | --- | --- |
| 0 | 1 | 2 | 3 | 4 | 5 |

1. Over the last year, how would you rate your **level** (degree) of discomfort or pain during or following vaginal penetration?

| Did not attempt intercourse | Very high | High | Moderate | Low | Very low or none at all |
| --- | --- | --- | --- | --- | --- |
| 0 | 1 | 2 | 3 | 4 | 5 |

FEMALE SEXUAL DISTRESS SCALE-REVISED (FSDS-R) (**FEMALE**)

Below is a list of feelings and problems that women sometimes have concerning their sexuality. Please read each item carefully, and circle the number that best describes **how** **often** that problem has bothered you or caused you distress **OVER THE LAST YEAR**. You may choose not to answer any question for any reason, and still return the questionnaire.

**Over the last year, how often did you feel**…

1. Distressed about your sex life

| Never | Rarely | Occasionally | Frequently | Always |
| --- | --- | --- | --- | --- |
| 0 | 1 | 2 | 3 | 4 |

1. Unhappy about your sexual relationship

| Never | Rarely | Occasionally | Frequently | Always |
| --- | --- | --- | --- | --- |
| 0 | 1 | 2 | 3 | 4 |

1. Guilty about sexual difficulties

| Never | Rarely | Occasionally | Frequently | Always |
| --- | --- | --- | --- | --- |
| 0 | 1 | 2 | 3 | 4 |

1. Frustrated by your sexual problems

| Never | Rarely | Occasionally | Frequently | Always |
| --- | --- | --- | --- | --- |
| 0 | 1 | 2 | 3 | 4 |

1. Stressed about sex

| Never | Rarely | Occasionally | Frequently | Always |
| --- | --- | --- | --- | --- |
| 0 | 1 | 2 | 3 | 4 |

1. Inferior because of sexual problems

| Never | Rarely | Occasionally | Frequently | Always |
| --- | --- | --- | --- | --- |
| 0 | 1 | 2 | 3 | 4 |

1. Worried about sex

| Never | Rarely | Occasionally | Frequently | Always |
| --- | --- | --- | --- | --- |
| 0 | 1 | 2 | 3 | 4 |

1. Sexually inadequate

| Never | Rarely | Occasionally | Frequently | Always |
| --- | --- | --- | --- | --- |
| 0 | 1 | 2 | 3 | 4 |

1. Regrets about your sexuality

| Never | Rarely | Occasionally | Frequently | Always |
| --- | --- | --- | --- | --- |
| 0 | 1 | 2 | 3 | 4 |

1. Embarrassed about sexual problems

| Never | Rarely | Occasionally | Frequently | Always |
| --- | --- | --- | --- | --- |
| 0 | 1 | 2 | 3 | 4 |

1. Dissatisfied with your sex life

| Never | Rarely | Occasionally | Frequently | Always |
| --- | --- | --- | --- | --- |
| 0 | 1 | 2 | 3 | 4 |

1. Angry about your sex life

| Never | Rarely | Occasionally | Frequently | Always |
| --- | --- | --- | --- | --- |
| 0 | 1 | 2 | 3 | 4 |

1. Bothered by low sexual desire

| Never | Rarely | Occasionally | Frequently | Always |
| --- | --- | --- | --- | --- |
| 0 | 1 | 2 | 3 | 4 |

SEXUAL HEALTH AND FUNCTION

You may choose not to answer any question for any reason, and still return the questionnaire.

Since the onset of hidradenitis suppurativa, has your sexual activity declined?

YES NO UNKNOWN

If YES, please rate the following factors according **to how often** they have affected your sexual functioning:

1. Hidradenitis suppurativa has a negative effect on my physical appearance

| Never | Sometimes | Often | Always |
| --- | --- | --- | --- |
| 1 | 2 | 3 | 4 |

1. My partner’s fear of contagiousness

| Never | Sometimes | Often | Always |
| --- | --- | --- | --- |
| 1 | 2 | 3 | 4 |

1. Fear of passing hidradenitis suppurative on to my children

| Never | Sometimes | Often | Always |
| --- | --- | --- | --- |
| 1 | 2 | 3 | 4 |

1. Diminished personal sexual desire

| Never | Sometimes | Often | Always |
| --- | --- | --- | --- |
| 1 | 2 | 3 | 4 |

1. Diminished sexual desire of my partner

| Never | Sometimes | Often | Always |
| --- | --- | --- | --- |
| 1 | 2 | 3 | 4 |

1. Inconvenience caused by skin lesions

| Never | Sometimes | Often | Always |
| --- | --- | --- | --- |
| 1 | 2 | 3 | 4 |

1. Inconvenience caused by topical treatments

| Never | Sometimes | Often | Always |
| --- | --- | --- | --- |
| 1 | 2 | 3 | 4 |

Is there sufficient attention given to possible sexual problems associated with hidradenitis suppurativa by healthcare professionals?

YES NO I’m Not Sure

Would you prefer more frequent questioning about possible sexual problems by healthcare professionals?

YES NO I’m Not Sure

**QUESTIONNAIRE – CONTROL GROUP**

**STUDY TITLE:** Quality of life and sexual health in patients with hidradenitis suppurativa

**SUBJECT CODE:**

Your responses will be kept completely confidential.

DEMOGRAPHIC INFORMATION:

1. Age (years):
2. Sex (M/F):
3. Height (cm):
4. Weight (kg):
5. Relationship status:

DERMATOLOGY LIFE QUALITY INDEX (DLQI):

The aim of this section of the questionnaire is to measure how much your skin condition has affected your life **OVER THE LAST YEAR**. Please check one box for each question. You may choose not to answer any question for any reason, and still return the questionnaire.

1. Over the last year, how **itchy, sore, painful, or stinging** has your skin been?

- Very much
- A lot
- A little
- Not at all

1. Over the last year, how **embarrassed or self-conscious** have you been because of your skin?

- Very much
- A lot
- A little
- Not at all

1. Over the last year, how much has your skin interfered with you going **shopping** or looking after your **home** or **garden**?

- Very much
- A lot
- A little
- Not at all
- Not relevant

1. Over the last year, how much has your skin influenced the **clothes** that you wear?

- Very much
- A lot
- A little
- Not at all
- Not relevant

1. Over the last year, how much has your skin affected any **social** or **leisure** activities?

- Very much
- A lot
- A little
- Not at all
- Not relevant

1. Over the last year, how much has your skin made it difficult for you to do any **sport**?

- Very much
- A lot
- A little
- Not at all
- Not relevant

1. Over the last year, has your skin prevented you from **working** or **studying**?

- Yes
- No
- Not relevant

If ‘No’, over the last year how much has your skin been a problem at **work** or **studying**?

- A lot
- A little
- Not at all

1. Over the last year, how much has your skin created problems with your **partner** or any of your **close friends** or **relatives**?

- Very much
- A lot
- A little
- Not at all
- Not relevant

1. Over the last year, how much has your skin caused **sexual difficulties**?

- Very much
- A lot
- A little
- Not at all
- Not relevant

1. Over the last year, how much of a problem has the **treatment** for your skin been, for example by making your home messy, or by taking up time?

- Very much
- A lot
- A little
- Not at all
- Not relevant

For the following section, please complete questionnaires for **males** or **females**, respectively.

In answering these questions, the following definitions apply:

Sexual activity can include caressing, foreplay, masturbation, and intercourse.

Sexual stimulation includes situations like foreplay with a partner, self-stimulation (masturbation), or sexual fantasy.

SEXUAL QUALITY OF LIFE (**MALE**)

This questionnaire consists of a set of questions, each asking about thoughts and feelings that you may have about your sex life. Please answer based on your thoughts and feelings **OVER THE LAST YEAR**. You may choose not to answer any question for any reason, and still return the questionnaire.

Please rate each one according to how much you agree or disagree with the statement, by circling **one** of six response choices.

1. When I think about my sexual life, I feel frustrated

| Completely Agree | Moderately Agree | Slightly Agree | Slightly Disagree | Moderately Disagree | Completely Disagree |
| --- | --- | --- | --- | --- | --- |
| 1 | 2 | 3 | 4 | 5 | 6 |

1. When I think about my sexual life, I feel depressed

| Completely Agree | Moderately Agree | Slightly Agree | Slightly Disagree | Moderately Disagree | Completely Disagree |
| --- | --- | --- | --- | --- | --- |
| 1 | 2 | 3 | 4 | 5 | 6 |

1. When I think about my sexual life, I feel like less of a man

| Completely Agree | Moderately Agree | Slightly Agree | Slightly Disagree | Moderately Disagree | Completely Disagree |
| --- | --- | --- | --- | --- | --- |
| 1 | 2 | 3 | 4 | 5 | 6 |

1. I have lost confidence in myself as a sexual partner

| Completely Agree | Moderately Agree | Slightly Agree | Slightly Disagree | Moderately Disagree | Completely Disagree |
| --- | --- | --- | --- | --- | --- |
| 1 | 2 | 3 | 4 | 5 | 6 |

1. When I think about my sexual life, I feel anxious

| Completely Agree | Moderately Agree | Slightly Agree | Slightly Disagree | Moderately Disagree | Completely Disagree |
| --- | --- | --- | --- | --- | --- |
| 1 | 2 | 3 | 4 | 5 | 6 |

1. When I think about my sexual life, I feel angry

| Completely Agree | Moderately Agree | Slightly Agree | Slightly Disagree | Moderately Disagree | Completely Disagree |
| --- | --- | --- | --- | --- | --- |
| 1 | 2 | 3 | 4 | 5 | 6 |

1. I worry about the future of my sexual life

| Completely Agree | Moderately Agree | Slightly Agree | Slightly Disagree | Moderately Disagree | Completely Disagree |
| --- | --- | --- | --- | --- | --- |
| 1 | 2 | 3 | 4 | 5 | 6 |

1. When I think about my sexual life, I am embarrassed

| Completely Agree | Moderately Agree | Slightly Agree | Slightly Disagree | Moderately Disagree | Completely Disagree |
| --- | --- | --- | --- | --- | --- |
| 1 | 2 | 3 | 4 | 5 | 6 |

1. When I think about my sexual life, I feel guilty

| Completely Agree | Moderately Agree | Slightly Agree | Slightly Disagree | Moderately Disagree | Completely Disagree |
| --- | --- | --- | --- | --- | --- |
| 1 | 2 | 3 | 4 | 5 | 6 |

1. When I think about my sexual life, I worry that my partner feels hurt or rejected

| Completely Agree | Moderately Agree | Slightly Agree | Slightly Disagree | Moderately Disagree | Completely Disagree |
| --- | --- | --- | --- | --- | --- |
| 1 | 2 | 3 | 4 | 5 | 6 |

1. When I think about my sexual life, I feel like I have lost something

| Completely Agree | Moderately Agree | Slightly Agree | Slightly Disagree | Moderately Disagree | Completely Disagree |
| --- | --- | --- | --- | --- | --- |
| 1 | 2 | 3 | 4 | 5 | 6 |

INTERNATIONAL INDEX OF ERECTILE FUNCTION (IIEF) (**MALE**)

The following questions ask about the effects your erection problems have had on your sex life **OVER THE LAST YEAR**. Please mark only one answer per question. You may choose not to answer any question for any reason, and still return the questionnaire.

1. Over the last year, how often were you able to get an erection during sexual activity?

| No sexual activity | Almost never or never | A few times (much less than half the time) | Sometimes (about half the time) | Most times (much more than half the time) | Almost always or always |
| --- | --- | --- | --- | --- | --- |
| 0 | 1 | 2 | 3 | 4 | 5 |

1. Over the last year, when you had erections with sexual stimulation, how often were your erections hard enough for penetration?

| No sexual activity | Almost never or never | A few times (much less than half the time) | Sometimes (about half the time) | Most times (much more than half the time) | Almost always or always |
| --- | --- | --- | --- | --- | --- |
| 0 | 1 | 2 | 3 | 4 | 5 |

1. Over the last year, when you attempted sexual intercourse, how often were you able to penetrate (enter) your partner?

| Did not attempt intercourse | Almost never or never | A few times (much less than half the time) | Sometimes (about half the time) | Most times (much more than half the time) | Almost always or always |
| --- | --- | --- | --- | --- | --- |
| 0 | 1 | 2 | 3 | 4 | 5 |

1. Over the last year, during sexual intercourse, **how often** were you able to maintain your erection after you had penetrated (entered) your partner?

| Did not attempt intercourse | Almost never or never | A few times (much less than half the time) | Sometimes (about half the time) | Most times (much more than half the time) | Almost always or always |
| --- | --- | --- | --- | --- | --- |
| 0 | 1 | 2 | 3 | 4 | 5 |

1. Over the last year, during sexual intercourse, **how difficult** was it to maintain your erection to completion of intercourse?

| Did not attempt intercourse | Extremely difficult | Very difficult | Difficult | Slightly difficult | Not difficult |
| --- | --- | --- | --- | --- | --- |
| 0 | 1 | 2 | 3 | 4 | 5 |

1. Over the last year, how many times have you attempted sexual intercourse?

| No attempts | One to two attempts | Three to four attempts | Five to six attempts | Seven to ten attempts | Eleven or more attempts |
| --- | --- | --- | --- | --- | --- |
| 0 | 1 | 2 | 3 | 4 | 5 |

1. Over the last year, when you attempted sexual intercourse, how often was it satisfactory for you?

| Did not attempt intercourse | Almost never or never | A few times (much less than half the time) | Sometimes (about half the time) | Most times (much more than half the time) | Almost always or always |
| --- | --- | --- | --- | --- | --- |
| 0 | 1 | 2 | 3 | 4 | 5 |

1. Over the last year, how much have you enjoyed sexual intercourse?

| No intercourse | No enjoyment | Not very enjoyable | Fairly enjoyable | Highly enjoyable | Very highly enjoyable |
| --- | --- | --- | --- | --- | --- |
| 0 | 1 | 2 | 3 | 4 | 5 |

1. Over the last year, when you had sexual stimulation **or** intercourse, how often did you ejaculate?

| No sexual stimulation or intercourse | Almost never or never | A few times (much less than half the time) | Sometimes (about half the time) | Most times (much more than half the time) | Almost always or always |
| --- | --- | --- | --- | --- | --- |
| 0 | 1 | 2 | 3 | 4 | 5 |

1. Over the last year, when you had sexual stimulation **or** intercourse, how often did you have the feeling of orgasm or climax?

| No sexual stimulation or intercourse | Almost never or never | A few times (much less than half the time) | Sometimes (about half the time) | Most times (much more than half the time) | Almost always or always |
| --- | --- | --- | --- | --- | --- |
| 0 | 1 | 2 | 3 | 4 | 5 |

1. Over the last year, how often have you felt sexual desire?

| Almost never or never | A few times (much less than half the time) | Sometimes (about half the time) | Most times (much more than half the time) | Almost always or always |
| --- | --- | --- | --- | --- |
| 1 | 2 | 3 | 4 | 5 |

1. Over the last year, how would you rate your level of sexual desire?

| Very low/none at all | Low | Moderate | High | Very high |
| --- | --- | --- | --- | --- |
| 1 | 2 | 3 | 4 | 5 |

1. Over the last year, how satisfied have you been with your overall **sex life**?

| Very dissatisfied | Moderately dissatisfied | About equally satisfied and dissatisfied | Moderately satisfied | Very satisfied |
| --- | --- | --- | --- | --- |
| 1 | 2 | 3 | 4 | 5 |

1. Over the last year, how satisfied have you been with you **sexual relationship** with your partner?

| Very dissatisfied | Moderately dissatisfied | About equally satisfied and dissatisfied | Moderately satisfied | Very satisfied |
| --- | --- | --- | --- | --- |
| 1 | 2 | 3 | 4 | 5 |

1. Over the last year, how would you rate your **confidence** that you could get and keep an erection?

| Very low | Low | Moderate | High | Very high |
| --- | --- | --- | --- | --- |
| 1 | 2 | 3 | 4 | 5 |

FEMALE SEXUAL FUNCTION INDEX (FSFI) (**FEMALE**)

This questionnaire consists of a set of questions, each asking about thoughts and feelings that you may have about your sex life. The statement may be positive or negative. Please answer based on your thoughts and feelings **OVER THE LAST YEAR**. You may choose not to answer any question for any reason, and still return the questionnaire.

Please rate each one according to how much you agree or disagree with the statement, by circling **one** of six response choices.

1. Over the last year, how **often** did you feel sexual desire or interest?

| Almost never or never | A few times (less than half the time) | Sometimes (about half the time) | Most times (more than half the time) | Almost always or always |
| --- | --- | --- | --- | --- |
| 1 | 2 | 3 | 4 | 5 |

1. Over the last year, how would you rate your **level** (degree) of sexual desire or interest?

| Very low or none at all | Low | Moderate | High | Very high |
| --- | --- | --- | --- | --- |
| 1 | 2 | 3 | 4 | 5 |

1. Over the last year, how **often** did you feel sexually aroused (“turned on”) during sexual activity or intercourse?

| No sexual activity | Almost never or never | A few times (less than half the time) | Sometimes (about half the time) | Most times (more than half the time) | Almost always or always |
| --- | --- | --- | --- | --- | --- |
| 0 | 1 | 2 | 3 | 4 | 5 |

1. Over the last year, how would you rate your **level** of sexual arousal (“turn on”) during sexual activity or intercourse?

| No sexual activity | Very low or none at all | Low | Moderate | High | Very high |
| --- | --- | --- | --- | --- | --- |
| 0 | 1 | 2 | 3 | 4 | 5 |

1. Over the last year, how **confident** were you about becoming sexually aroused during sexual activity or intercourse?

| No sexual activity | Very low or no confidence | Low confidence | Moderate confidence | High confidence | Very high confidence |
| --- | --- | --- | --- | --- | --- |
| 0 | 1 | 2 | 3 | 4 | 5 |

1. Over the last year, how **often** have you been satisfied with your arousal (excitement) during sexual activity or intercourse?

| No sexual activity | Almost never or never | A few times (less than half the time) | Sometimes (about half the time) | Most times (more than half the time) | Almost always or always |
| --- | --- | --- | --- | --- | --- |
| 0 | 1 | 2 | 3 | 4 | 5 |

1. Over the last year, how **often** did you become lubricated (“wet”) during sexual activity or intercourse?

| No sexual activity | Almost never or never | A few times (less than half the time) | Sometimes (about half the time) | Most times (more than half the time) | Almost always or always |
| --- | --- | --- | --- | --- | --- |
| 0 | 1 | 2 | 3 | 4 | 5 |

1. Over the last year, how **difficult** was it to become lubricated (“wet”) during sexual activity or intercourse?

| No sexual activity | Extremely difficult or impossible | Very difficult | Difficult | Slightly difficult | Not difficult |
| --- | --- | --- | --- | --- | --- |
| 0 | 1 | 2 | 3 | 4 | 5 |

1. Over the last year, how **often** did you maintain your lubrication (“wetness”) until completion of sexual activity or intercourse?

| No sexual activity | Almost never or never | A few times (less than half the time) | Sometimes (about half the time) | Most times (more than half the time) | Almost always or always |
| --- | --- | --- | --- | --- | --- |
| 0 | 1 | 2 | 3 | 4 | 5 |

1. Over the last year, how **difficult** was it to maintain your lubrication (“wetness”) until completion of sexual activity or intercourse?

| No sexual activity | Extremely difficult or impossible | Very difficult | Difficult | Slightly difficult | Not difficult |
| --- | --- | --- | --- | --- | --- |
| 0 | 1 | 2 | 3 | 4 | 5 |

1. Over the last year, when you had sexual stimulation or intercourse, how **often** did you reach orgasm (climax)?

| No sexual activity | Almost never or never | A few times (less than half the time) | Sometimes (about half the time) | Most times (more than half the time) | Almost always or always |
| --- | --- | --- | --- | --- | --- |
| 0 | 1 | 2 | 3 | 4 | 5 |

1. Over the last year, when you had sexual stimulation or intercourse, how **difficult** was it for you to reach orgasm (climax)?

| No sexual activity | Extremely difficult or impossible | Very difficult | Difficult | Slightly difficult | Not difficult |
| --- | --- | --- | --- | --- | --- |
| 0 | 1 | 2 | 3 | 4 | 5 |

1. Over the last year, how **satisfied** were you with your ability to reach orgasm (climax) during sexual activity or intercourse?

| No sexual activity | Very dissatisfied | Moderately dissatisfied | About equally satisfied and dissatisfied | Moderately satisfied | Very satisfied |
| --- | --- | --- | --- | --- | --- |
| 0 | 1 | 2 | 3 | 4 | 5 |

1. Over the last year, how **satisfied** have you been with the amount of emotional closeness during sexual activity between you and your partner?

| No sexual activity | Very dissatisfied | Moderately dissatisfied | About equally satisfied and dissatisfied | Moderately satisfied | Very satisfied |
| --- | --- | --- | --- | --- | --- |
| 0 | 1 | 2 | 3 | 4 | 5 |

1. Over the last year, how satisfied have you been with your sexual relationship with your partner?

| Very dissatisfied | Moderately dissatisfied | About equally satisfied and dissatisfied | Moderately satisfied | Very satisfied |
| --- | --- | --- | --- | --- |
| 1 | 2 | 3 | 4 | 5 |

1. Over the last year, how **satisfied** have you been with your overall sexual life?

| Very dissatisfied | Moderately dissatisfied | About equally satisfied and dissatisfied | Moderately satisfied | Very satisfied |
| --- | --- | --- | --- | --- |
| 1 | 2 | 3 | 4 | 5 |

1. Over the last year, how **often** did you experience discomfort or pain **during** vaginal penetration?

| Did not attempt intercourse | Almost always or always | Most times (more than half the time) | Sometimes (about half the time) | A few times (less than half the time) | Almost never or never |
| --- | --- | --- | --- | --- | --- |
| 0 | 1 | 2 | 3 | 4 | 5 |

1. Over the last year, how **often** did you experience discomfort or pain **following** vaginal penetration?

| Did not attempt intercourse | Almost always or always | Most times (more than half the time) | Sometimes (about half the time) | A few times (less than half the time) | Almost never or never |
| --- | --- | --- | --- | --- | --- |
| 0 | 1 | 2 | 3 | 4 | 5 |

1. Over the last year, how would you rate your **level** (degree) of discomfort or pain during or following vaginal penetration?

| Did not attempt intercourse | Very high | High | Moderate | Low | Very low or none at all |
| --- | --- | --- | --- | --- | --- |
| 0 | 1 | 2 | 3 | 4 | 5 |

FEMALE SEXUAL DISTRESS SCALE-REVISED (FSDS-R) (**FEMALE**)

Below is a list of feelings and problems that women sometimes have concerning their sexuality. Please read each item carefully, and circle the number that best describes **how** **often** that problem has bothered you or caused you distress **OVER THE LAST YEAR**. You may choose not to answer any question for any reason, and still return the questionnaire.

**Over the last year, how often did you feel**…

1. Distressed about your sex life

| Never | Rarely | Occasionally | Frequently | Always |
| --- | --- | --- | --- | --- |
| 0 | 1 | 2 | 3 | 4 |

1. Unhappy about your sexual relationship

| Never | Rarely | Occasionally | Frequently | Always |
| --- | --- | --- | --- | --- |
| 0 | 1 | 2 | 3 | 4 |

1. Guilty about sexual difficulties

| Never | Rarely | Occasionally | Frequently | Always |
| --- | --- | --- | --- | --- |
| 0 | 1 | 2 | 3 | 4 |

1. Frustrated by your sexual problems

| Never | Rarely | Occasionally | Frequently | Always |
| --- | --- | --- | --- | --- |
| 0 | 1 | 2 | 3 | 4 |

1. Stressed about sex

| Never | Rarely | Occasionally | Frequently | Always |
| --- | --- | --- | --- | --- |
| 0 | 1 | 2 | 3 | 4 |

1. Inferior because of sexual problems

| Never | Rarely | Occasionally | Frequently | Always |
| --- | --- | --- | --- | --- |
| 0 | 1 | 2 | 3 | 4 |

1. Worried about sex

| Never | Rarely | Occasionally | Frequently | Always |
| --- | --- | --- | --- | --- |
| 0 | 1 | 2 | 3 | 4 |

1. Sexually inadequate

| Never | Rarely | Occasionally | Frequently | Always |
| --- | --- | --- | --- | --- |
| 0 | 1 | 2 | 3 | 4 |

1. Regrets about your sexuality

| Never | Rarely | Occasionally | Frequently | Always |
| --- | --- | --- | --- | --- |
| 0 | 1 | 2 | 3 | 4 |

1. Embarrassed about sexual problems

| Never | Rarely | Occasionally | Frequently | Always |
| --- | --- | --- | --- | --- |
| 0 | 1 | 2 | 3 | 4 |

1. Dissatisfied with your sex life

| Never | Rarely | Occasionally | Frequently | Always |
| --- | --- | --- | --- | --- |
| 0 | 1 | 2 | 3 | 4 |

1. Angry about your sex life

| Never | Rarely | Occasionally | Frequently | Always |
| --- | --- | --- | --- | --- |
| 0 | 1 | 2 | 3 | 4 |

1. Bothered by low sexual desire

| Never | Rarely | Occasionally | Frequently | Always |
| --- | --- | --- | --- | --- |
| 0 | 1 | 2 | 3 | 4 |

SEXUAL HEALTH AND FUNCTION

Would you prefer more frequent questioning about possible sexual problems by healthcare professionals?

YES NO I’m Not Sure
